# 6-[Bis(ethoxycarbonyl)methyl]-6-deoxy-1,2;3,4-di-*O*-isopropyl­idene-d-galacto­pyran­ose

**DOI:** 10.1107/S1600536810046921

**Published:** 2010-11-17

**Authors:** Bogdan Doboszewski, Paulo R. da Silva, Alexander Y. Nazarenko, Victor N. Nemykin

**Affiliations:** a Departamento de Química, Universidade Federal Rural de Pernambuco, 52171-900 Recife, PE, Brazil; b Chemistry Department, State University of New York, College at Buffalo, 1300 Elmwood Ave, Buffalo, NY 14222-1095, USA; c Department of Chemistry & Biochemistry, University of Minnesota Duluth, Duluth, Minnesota 55812-2496, USA

## Abstract

The title compound, C_19_H_30_O_9_, was prepared by substitution at the C6 position in 1,2;3,4-di-*O*-isopropyl­idene-6-*O*-trifluoro­methane­sulfonyl-d-galactose using sodium eth­oxy­malonate in dimethyl­formamide. The conformation is skew-boat ^0^ *S*_2_, slightly distorted towards boat *B*_2,5_. The inflexible pyran­ose structure makes the title compound a suitable inter­mediate for further synthetic work by keeping stereogenic carbon atoms safe from inversion. Several short intra­molecular C—H⋯ O contacts may stabilize the conformation of the mol­ecule. Inter­molecular C—H⋯O inter­actions also occur.

## Related literature

For syntheses of this and similar compounds, see: Bouhlal *et al.* (2001 ▶); Doboszewski *et al.* (1987 ▶); Honeyman & Stening (1958 ▶); Sugihara *et al.* (1963 ▶); Tipson (1953 ▶); Cipolla *et al.* (1996 ▶). For the structures of diisopropyl­idene-galactopyran­ose and related compounds, see: Krajewski *et al.* (1990 ▶, 1994 ▶); Coutrot *et al.* (2001 ▶); Weaver *et al.* (2004 ▶, 2006 ▶); Boeyens *et al.* (1978 ▶); Berces *et al.* (2001 ▶). For conformations of small rings, see: Schwarz (1973 ▶); Cremer & Pople (1975 ▶); Boeyens (1978 ▶); Hill & Reilly (2007 ▶); Köll *et al.* (1994 ▶). For analysis of absolute structure, see: Flack (1983 ▶); Hooft *et al.* (2008 ▶).
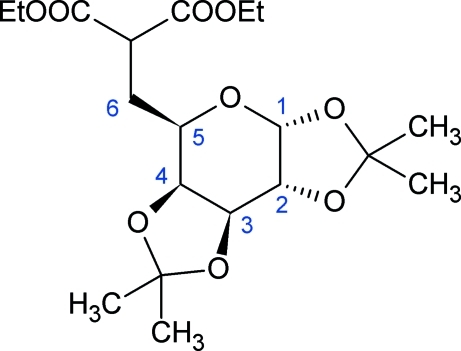


## Experimental

### 

#### Crystal data

C_19_H_30_O_9_
*M* *_r_* = 402.43
Orthorhombic, 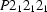

*a* = 8.3287 (4) Å
*b* = 10.8895 (4) Å
*c* = 23.7706 (16) Å
*V* = 2155.9 (2) Å^3^
*Z* = 4
Cu *K*α radiation
μ = 0.83 mm^−1^
*T* = 292 K
0.38 × 0.26 × 0.21 mm

#### Data collection

Rigaku R-AXIS RAPID II imaging plate diffractometer
Absorption correction: multi-scan (*ABSCOR*; Higashi,1995 ▶) *T*_min_ = 0.822, *T*_max_ = 0.840
9353 measured reflections
3745 independent reflections
2824 reflections with *I* > 2σ(*I*)
*R*_int_ = 0.075

#### Refinement

*R*[*F* ^2^ > 2σ(*F* ^2^)] = 0.040
*wR*(*F* ^2^) = 0.096
*S* = 1.02
3745 reflections
275 parameters
H atoms treated by a mixture of independent and constrained refinement
Δρ_max_ = 0.19 e Å^−3^
Δρ_min_ = −0.15 e Å^−3^
Absolute structure: Flack (1983 ▶), 1607 Friedel pairs
Flack parameter: 0.06 (18)

### 

Data collection: *CrystalClear-SM Expert* (Rigaku, 2009 ▶); cell refinement: *HKL-2000* (Otwinowski & Minor, 1997 ▶); data reduction: *CrystalClear-SM Expert*; program(s) used to solve structure: *SHELXS97* (Sheldrick, 2008 ▶); program(s) used to refine structure: *SHELXL97* (Sheldrick, 2008 ▶); molecular graphics: *ORTEP-3 for Windows* (Farrugia, 1997 ▶); software used to prepare material for publication: *PLATON* (Spek, 2009) ▶.

## Supplementary Material

Crystal structure: contains datablocks global, I. DOI: 10.1107/S1600536810046921/zl2327sup1.cif

Structure factors: contains datablocks I. DOI: 10.1107/S1600536810046921/zl2327Isup2.hkl

Additional supplementary materials:  crystallographic information; 3D view; checkCIF report

## Figures and Tables

**Table 1:** Hydrogen-bond geometry (Å, °)

*D*—H⋯*A*	*D*—H	H⋯*A*	*D*⋯*A*	*D*—H⋯*A*
C1—H1*A*⋯O9^i^	0.98	2.43	3.381 (3)	163
C5—H5*A*⋯O7	1.01	2.59	3.185 (3)	117
C8—H8*B*⋯O7^i^	1.03	2.56	3.577 (3)	169
C12—H12*A*⋯O5	1.02	2.42	2.811 (3)	102
C13—H13*A*⋯O1	0.96	2.43	2.814 (3)	103
C16—H16*B*⋯O1^ii^	0.99	2.51	3.422 (3)	153

Symmetry codes: (i) 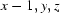; (ii) 
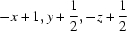
.

**Fig. 1.**
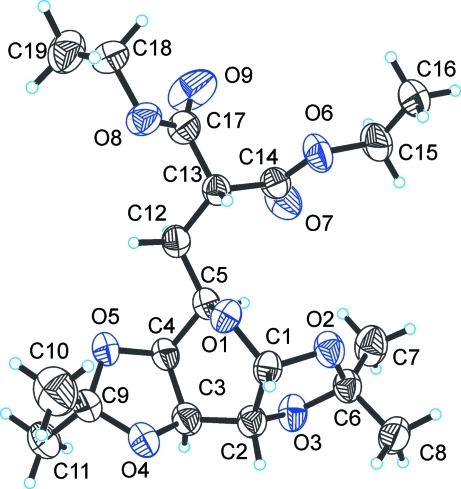
The title compound with displacement ellipsoids drawn at the 50% probability level. H atoms are shown as spheres of arbitary radius.

**Fig. 2.**
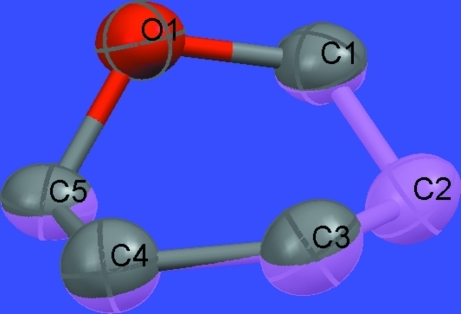
Conformation of the six-membered ring: mean plane drawn through C1—C3—C4—C5.

**Fig. 3.**
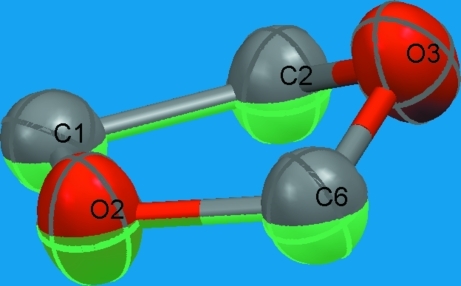
Conformation of the five-membered isopropylidene ring: plane through C1 C2 C6 O2.

**Fig. 4.**
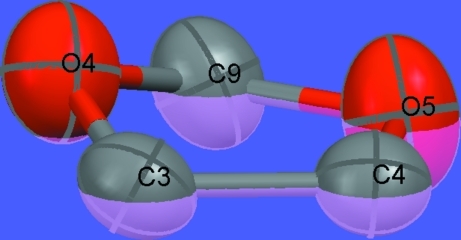
Conformation of the five-membered isopropylidene ring: plane through C3 C9 O5 C4.

**Fig. 5.**
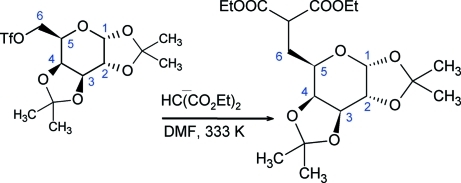
Synthetic route towards the title compound.

## supplementary crystallographic information

### Comment

The title compound is an intermediate for the synthesis of a wide range of chain-extended galactopyranoses, which in turn are considered as precursors of chiral α-hydroxycarboxylic acids. The stereogenic atom C5 is retained in the target compounds. Knowledge of exact geometry of the intermediate is helpful for better understanding of upcoming steps in this ongoing synthetic project.

To realise these objectives we (BD & PRS) have prepared the title compound by substitution at the C6 position in 1,2;3,4-di-*O*-isopropylidene-6-*O*-trifluoromethanesulfonyl-D-galactose using sodium ethoxymalonate. Substitutions at this atom in diisopropylidene-galactose are rather difficult and require prolonged reaction times and/or elevated temperatures (Tipson, 1953, Honeyman & Stening, 1958, Sugihara *et al.*, 1963, Bouhlal *et al.*, 2001). Howeever, by using the best available leaving group, a trifluoromethanesulfonate, smooth nucleophilic substitution can been accomplished in less than 10 min (Doboszewski *et al.*, 1987).

The absolute structure of 6-deoxy-6-(diethylmalonyl)-1,2;3,4-di-*O* -isopropylidene-D-galactopyranose is certain from the synthetic route which does not affect asymmetric atoms of the starting compound. Nevertheless, we preferred to receive a direct experimental confirmation using X-ray diffractometry data. Because there are no heavy atoms in a chiral molecule of title compound, Cu *K*α radiation was necessary for determination of the absolute structure.

In the crystal structure of title compound (Fig.1), all bond lengths and bond angles have standard dimensions.

Fig. 2 shows that the pyranose ring adopts a skew-boat with atoms C1, C3, C4, and C5 being within 0.03–0.08 Å from their mean plane, and O1 and C2 atoms being at 0.633 (2) and -0.557 (2) Å, respectively. Such conformation is named ^O^S_2_ in the IUPAC notation (Schwarz, 1973). A quantitative analysis of the ring conformations in the titlw compound was performed using the method of Cremer and Pople (Cremer & Pople, 1975, Boeyens, 1978) for the calculation of parameters of puckering. The polar parameters for the pyranose ring are Q = 0.639 (2) Å, Φ = 325.1 (2)°, and θ = 80.3 (2)°. These suggest the conformation as skew-boat ^0^S_2_ (Φ = 330°, θ = 90°), slightly distorted towards boat B_2,5_, (Φ = 300°, θ = 90°); the same conformation is designated as a twist-boat ^O^T_2_ when using the Boeyenes nomenclature. This conformation is similar to many other known galactopyranoses with two substituent isopropylidene rings (see, for example, POCSUV (Krajewski *et al.*, 1994): Q = 0.632 (5) Å, θ = 82.8 (5)°,Φ = 327.4 (4)°; JERJIUL (Krajewski *et al.*, 1990): Q = 0.631 (5) Å, θ = 79.7 (5)°, Φ = 324.9 (5)°; ICALED (Coutrot *et al.*, 2001): Q = 0.646 (4) Å, θ = 83.9 (4)°, Φ = 334.2 (3)°; BIHZUO (Weaver *et al.*, 2004): Q = 0.661 (2) Å, θ = 81.3 (2)°, Φ = 327.1 (2)°; ADXPOP (Boeyens, Rathbone & Woolard,1978): Q = 0.65 Å, Φ = 329°, θ = 81°). All conformations of substituted compounds are radically different from the chair conformation of unsubstituted α -D-galactopyranose. This is caused by the presence of the two isopropylidene substituents that make the geometry of the pyranose ring more rigid and less sensitive towards any effects of substituents at the remaining C5 position. A detailed discussion of terminology and different puckering coordinates being used to describe six-membered non-aromatic cycles can be found in Hill & Reilly (2007) and Köll *et al.* (1994).

The same approach yielded the parameters of puckering Q(2) = 0.279 (2) Å, Φ = 283.3 (4)° and Q(2) = 0.234 (2) Å, Φ = 177.1 (7)° for the 1,2- and 3,4- isopropylidene rings. These values correspond to the envelope conformations ^4^E (Φ = 288°) and E_1_ (Φ = 180°) with atoms O3 and O4 being out of their corresponding planes by 0.426 (2) and 0.357 (2) Å correspondingly (Fig. 3 and 4). All other atoms in both five-membered rings are located within 0.01 Å from their mean planes.

No classic hydrogen bonds are possible for the title compound. However, several short C—H··· O contacts were detected that possibly stabilize the existing conformation of the molecule (Table 1).

The inflexible pyranose structure makes the title compound a suitable intermediate for further synthetic work by keeping the stereogenic carbon atoms C1—C5 safe from inversion. For the same reason, it is very probable that in solution this molecule will keep almost the same geometry as in the molecular crystal.

### Experimental

Synthesis of the title compound was accomplished similar to previuosly published fluorination reaction (Doboszewski *et al.*, 1987). We treated 1,2;3,4-di-*O*-isopropylidene-6-*O*-trifluoromethanesulfonyl-D- galactose with sodium ethoxymalonate in dimethylformamide at 333 K; the title compound was isolated in 80% yield (Figure 5). The compound is identical to previously obtained  *via* a free-radical  process in low yield (Cipolla *et al.*, 1996). Spontaneous crystallization from a hexane-ethyl acetate system yielded colourless crystals suitable for single-crystal diffractometry (m.p. 331–334 K).

### Refinement

The chirality of the title compound was known from the synthetic route; it was also examined using anomalous scattering. Analysis of the absolute structure using likelihood methods (Hooft *et al.*, 2008) was performed using *PLATON* (Spek, 2003); 1570 Bijvoet pairs were employed. The results confirmed that the absolute structure had been correctly assigned: the probability that the structure is inverted is smaller than 10^-9^ with probability of racemic twinning at 0.002. Because no atom heavier than O is present, the standard deviation of the Flack parameter is relatively high. All H atoms were positioned geometrically and refined using a riding model, with C–H = 0.99–1.03 Å and *U*_iso_(H) = 1.2 or 1.5 *U*_eq_(C). The rotating group model was applied for the methyl groups.

### Crystal data

**Table:**

C_19_H_30_O_9_	*D*_x_ = 1.240 Mg m^−^^3^
*M**_r_* = 402.43	Melting point: 322 K
Orthorhombic, *P*2_1_2_1_2_1_	Cu *K*α radiation, λ = 1.54187 Å
Hall symbol: P 2ac 2ab	Cell parameters from 9579 reflections
*a* = 8.3287 (4) Å	θ = 6.7–68.2°
*b* = 10.8895 (4) Å	µ = 0.83 mm^−^^1^
*c* = 23.7706 (16) Å	*T* = 292 K
*V* = 2155.9 (2) Å^3^	Prism, colourless
*Z* = 4	0.38 × 0.26 × 0.21 mm
*F*(000) = 864	

### Data collection

**Table:**

Rigaku R-AXIS RAPID II imaging plate diffractometer	3745 independent reflections
Radiation source: fine-focus sealed tube	2824 reflections with *I* > 2σ(*I*)
graphite	*R*_int_ = 0.075
Detector resolution: 10 pixels mm^-1^	θ_max_ = 66.5°, θ_min_ = 6.7°
ω scans	*h* = −6→9
Absorption correction: multi-scan (*ABSCOR*; Higashi,1995)	*k* = −12→10
*T*_min_ = 0.822, *T*_max_ = 0.840	*l* = −28→19
9353 measured reflections	

### Refinement

**Table:**

Refinement on *F*^2^	Hydrogen site location: inferred from neighbouring sites
Least-squares matrix: full	H atoms treated by a mixture of independent and constrained refinement
*R*[*F*^2^ > 2σ(*F*^2^)] = 0.040	*w* = 1/[σ^2^(*F*_o_^2^) + (0.0285*P*)^2^] where *P* = (*F*_o_^2^ + 2*F*_c_^2^)/3
*wR*(*F*^2^) = 0.096	(Δ/σ)_max_ < 0.001
*S* = 1.02	Δρ_max_ = 0.19 e Å^−^^3^
3745 reflections	Δρ_min_ = −0.15 e Å^−^^3^
275 parameters	Extinction correction: *SHELXL*, Fc^*^=kFc[1+0.001xFc^2^λ^3^/sin(2θ)]^-1/4^
0 restraints	Extinction coefficient: 0.0057 (5)
Primary atom site location: structure-invariant direct methods	Absolute structure: Flack (1983), 1607 Friedel pairs
Secondary atom site location: difference Fourier map	Flack parameter: 0.06 (18)

### Special details

**Table:**

Geometry. All e.s.d.'s (except the e.s.d. in the dihedral angle between two l.s. planes) are estimated using the full covariance matrix. The cell e.s.d.'s are taken into account individually in the estimation of e.s.d.'s in distances, angles and torsion angles; correlations between e.s.d.'s in cell parameters are only used when they are defined by crystal symmetry. An approximate (isotropic) treatment of cell e.s.d.'s is used for estimating e.s.d.'s involving l.s. planes.
Refinement. Refinement of *F*^2^ against ALL reflections. The weighted *R*-factor *wR* and goodness of fit *S* are based on *F*^2^, conventional *R*-factors *R* are based on *F*, with *F* set to zero for negative *F*^2^. The threshold expression of *F*^2^ > σ(*F*^2^) is used only for calculating *R*-factors(gt) *etc*. and is not relevant to the choice of reflections for refinement. *R*-factors based on *F*^2^ are statistically about twice as large as those based on *F*, and *R*- factors based on ALL data will be even larger.

### Fractional atomic coordinates and isotropic or equivalent isotropic displacement parameters (Å^2^)

**Table:**

	*x*	*y*	*z*	*U*_iso_*/*U*_eq_	
O1	0.30842 (18)	0.05824 (11)	0.37716 (6)	0.0531 (4)	
O2	0.2263 (2)	0.25329 (12)	0.35151 (6)	0.0653 (5)	
O3	0.2044 (2)	0.30221 (12)	0.44385 (6)	0.0646 (5)	
O4	0.0989 (2)	−0.00622 (15)	0.48415 (7)	0.0747 (5)	
O5	0.3563 (2)	−0.07330 (14)	0.48515 (7)	0.0817 (6)	
O6	0.6685 (2)	0.16820 (13)	0.27264 (7)	0.0718 (5)	
O7	0.7237 (2)	0.22413 (15)	0.36113 (7)	0.0852 (6)	
O8	0.7062 (2)	−0.17738 (13)	0.31233 (6)	0.0666 (5)	
O9	0.8976 (2)	−0.03812 (18)	0.32008 (11)	0.1155 (9)	
C1	0.1819 (3)	0.14252 (17)	0.37891 (9)	0.0523 (6)	
H1A	0.088 (2)	0.1070 (8)	0.3597 (4)	0.063*	
C2	0.1329 (3)	0.18443 (17)	0.43770 (9)	0.0573 (6)	
H2A	0.012 (3)	0.1918 (2)	0.43999 (11)	0.069*	
C3	0.1939 (3)	0.10213 (19)	0.48485 (10)	0.0600 (6)	
H3A	0.1836 (4)	0.1456 (10)	0.5227 (8)	0.072*	
C4	0.3665 (3)	0.0557 (2)	0.47632 (9)	0.0566 (6)	
H4A	0.4367 (16)	0.0921 (8)	0.5044 (6)	0.068*	
C5	0.4315 (3)	0.0817 (2)	0.41783 (9)	0.0490 (5)	
H5A	0.4630 (7)	0.1708 (18)	0.41554 (10)	0.059*	
C6	0.2124 (3)	0.35512 (18)	0.38911 (9)	0.0580 (6)	
C7	0.3592 (3)	0.4335 (2)	0.38497 (12)	0.0860 (10)	
H7A	0.3572 (11)	0.4945 (14)	0.4142 (7)	0.129*	
H7B	0.3615 (12)	0.4733 (15)	0.3489 (6)	0.129*	
H7C	0.4532 (15)	0.3831 (8)	0.3892 (8)	0.129*	
C8	0.0607 (3)	0.4254 (2)	0.37542 (11)	0.0742 (8)	
H8A	0.0457 (11)	0.4951 (13)	0.4040 (6)	0.111*	
H8B	−0.0360 (13)	0.3671 (8)	0.3773 (7)	0.111*	
H8C	0.0694 (9)	0.4617 (14)	0.3357 (6)	0.111*	
C9	0.1971 (3)	−0.1063 (2)	0.49989 (10)	0.0657 (7)	
C10	0.1486 (5)	−0.2177 (2)	0.46606 (14)	0.1124 (13)	
H10A	0.034 (2)	−0.2405 (13)	0.4754 (7)	0.169*	
H10B	0.222 (2)	−0.2886 (14)	0.4756 (8)	0.169*	
H10C	0.157 (3)	−0.1985 (8)	0.4246 (7)	0.169*	
C11	0.1883 (4)	−0.1306 (3)	0.56235 (11)	0.0901 (10)	
H11A	0.0735 (17)	−0.1545 (17)	0.5730 (2)	0.135*	
H11B	0.221 (2)	−0.0531 (13)	0.5838 (3)	0.135*	
H11C	0.265 (2)	−0.2007 (15)	0.5725 (2)	0.135*	
C12	0.5762 (3)	0.00309 (19)	0.40257 (9)	0.0557 (6)	
H12A	0.5494 (4)	−0.0868 (13)	0.40954 (15)	0.067*	
H12B	0.6699 (14)	0.0260 (3)	0.4279 (4)	0.067*	
C13	0.6261 (3)	0.01977 (16)	0.34089 (9)	0.0510 (6)	
H13A	0.535 (2)	0.0017 (4)	0.3179 (5)	0.061*	
C14	0.6788 (3)	0.1493 (2)	0.32781 (11)	0.0585 (6)	
C15	0.7163 (4)	0.2883 (2)	0.25156 (13)	0.0870 (10)	
H15A	0.826 (2)	0.3111 (5)	0.2665 (3)	0.104*	
H15B	0.6368 (15)	0.3532 (13)	0.2641 (3)	0.104*	
C16	0.7196 (4)	0.2807 (2)	0.18966 (12)	0.0887 (10)	
H16A	0.799 (2)	0.2184 (16)	0.1779 (2)	0.133*	
H16B	0.750 (2)	0.3614 (13)	0.1739 (3)	0.133*	
H16C	0.6122 (18)	0.2572 (17)	0.1757 (3)	0.133*	
C17	0.7604 (3)	−0.0665 (2)	0.32388 (10)	0.0608 (6)	
C18	0.8256 (3)	−0.2666 (2)	0.29360 (12)	0.0725 (8)	
H18A	0.9058 (14)	−0.2830 (3)	0.3248 (5)	0.087*	
H18B	0.8859 (11)	−0.2336 (6)	0.2598 (5)	0.087*	
C19	0.7401 (4)	−0.3825 (2)	0.27835 (11)	0.0828 (10)	
H19A	0.6860 (18)	−0.4159 (10)	0.3119 (5)	0.124*	
H19B	0.8182 (11)	−0.4430 (10)	0.2641 (7)	0.124*	
H19C	0.6597 (18)	−0.3651 (4)	0.2490 (6)	0.124*	

### Atomic displacement parameters (Å^2^)

**Table:**

	*U*^11^	*U*^22^	*U*^33^	*U*^12^	*U*^13^	*U*^23^
O1	0.0620 (10)	0.0461 (7)	0.0511 (9)	0.0023 (8)	−0.0101 (7)	−0.0076 (7)
O2	0.0976 (13)	0.0470 (8)	0.0515 (9)	0.0008 (8)	0.0044 (9)	0.0014 (6)
O3	0.0955 (13)	0.0472 (8)	0.0510 (9)	0.0018 (9)	−0.0093 (9)	−0.0041 (7)
O4	0.0744 (13)	0.0687 (10)	0.0811 (13)	−0.0030 (10)	0.0129 (9)	0.0232 (9)
O5	0.0809 (13)	0.0615 (9)	0.1028 (14)	0.0083 (10)	0.0273 (11)	0.0349 (9)
O6	0.0982 (15)	0.0526 (8)	0.0645 (11)	−0.0142 (9)	0.0074 (10)	0.0135 (8)
O7	0.1065 (16)	0.0676 (10)	0.0814 (13)	−0.0359 (11)	0.0086 (12)	−0.0092 (9)
O8	0.0630 (11)	0.0515 (8)	0.0854 (12)	−0.0037 (9)	0.0082 (10)	−0.0091 (8)
O9	0.0547 (12)	0.0826 (12)	0.209 (3)	−0.0130 (11)	0.0071 (14)	−0.0325 (14)
C1	0.0597 (15)	0.0464 (11)	0.0508 (13)	−0.0018 (12)	−0.0106 (12)	−0.0010 (9)
C2	0.0730 (18)	0.0486 (12)	0.0504 (14)	0.0037 (12)	0.0039 (12)	−0.0024 (10)
C3	0.0764 (18)	0.0570 (12)	0.0466 (14)	0.0017 (14)	0.0079 (13)	0.0032 (10)
C4	0.0679 (17)	0.0551 (12)	0.0467 (13)	−0.0024 (13)	−0.0017 (12)	0.0068 (11)
C5	0.0588 (15)	0.0430 (10)	0.0452 (13)	−0.0078 (11)	−0.0057 (11)	0.0016 (9)
C6	0.0759 (18)	0.0446 (11)	0.0534 (14)	0.0025 (13)	−0.0021 (13)	0.0002 (10)
C7	0.087 (2)	0.0612 (15)	0.110 (2)	−0.0130 (16)	−0.0115 (17)	0.0115 (15)
C8	0.0827 (19)	0.0598 (14)	0.0800 (19)	0.0081 (15)	−0.0057 (14)	0.0083 (13)
C9	0.0756 (19)	0.0581 (13)	0.0635 (16)	0.0010 (14)	0.0195 (15)	0.0125 (11)
C10	0.146 (4)	0.0824 (19)	0.108 (3)	−0.020 (2)	0.013 (2)	−0.0156 (18)
C11	0.109 (2)	0.0972 (19)	0.0644 (17)	0.015 (2)	0.0238 (17)	0.0259 (15)
C12	0.0600 (15)	0.0517 (11)	0.0555 (15)	−0.0035 (13)	−0.0012 (12)	0.0091 (10)
C13	0.0539 (14)	0.0458 (11)	0.0534 (14)	−0.0058 (11)	0.0009 (11)	0.0032 (10)
C14	0.0595 (17)	0.0515 (12)	0.0645 (16)	−0.0050 (12)	0.0059 (13)	0.0027 (11)
C15	0.113 (3)	0.0524 (13)	0.096 (2)	−0.0125 (16)	0.026 (2)	0.0189 (14)
C16	0.091 (2)	0.0784 (17)	0.096 (2)	0.0061 (17)	0.0249 (19)	0.0356 (16)
C17	0.0568 (17)	0.0571 (13)	0.0684 (16)	−0.0068 (13)	0.0037 (13)	−0.0003 (12)
C18	0.0680 (19)	0.0702 (15)	0.0794 (18)	0.0135 (16)	0.0072 (15)	−0.0077 (13)
C19	0.109 (3)	0.0585 (14)	0.0807 (19)	0.0077 (16)	−0.0041 (17)	−0.0091 (13)

### Geometric parameters (Å, °)

**Table:**

O1—C1	1.398 (2)	C7—H7C	0.9614
O1—C5	1.432 (2)	C8—H8A	1.0264
O2—C1	1.420 (2)	C8—H8B	1.0264
O2—C6	1.429 (2)	C8—H8C	1.0264
O3—C2	1.422 (2)	C9—C11	1.510 (3)
O3—C6	1.425 (2)	C9—C10	1.511 (4)
O4—C9	1.413 (3)	C10—H10A	1.0094
O4—C3	1.421 (2)	C10—H10B	1.0094
O5—C9	1.418 (3)	C10—H10C	1.0094
O5—C4	1.423 (3)	C11—H11A	1.0229
O6—C14	1.330 (3)	C11—H11B	1.0229
O6—C15	1.456 (2)	C11—H11C	1.0229
O7—C14	1.196 (3)	C12—C13	1.535 (3)
O8—C17	1.317 (3)	C12—H12A	1.0171
O8—C18	1.460 (3)	C12—H12B	1.0171
O9—C17	1.187 (3)	C13—C14	1.509 (3)
C1—C2	1.526 (3)	C13—C17	1.515 (3)
C1—H1A	0.9878	C13—H13A	0.9571
C2—C3	1.523 (3)	C15—C16	1.474 (4)
C2—H2A	1.0091	C15—H15A	1.0130
C3—C4	1.537 (3)	C15—H15B	1.0130
C3—H3A	1.0200	C16—H16A	0.9879
C4—C5	1.518 (3)	C16—H16B	0.9879
C4—H4A	0.9713	C16—H16C	0.9879
C5—C12	1.522 (3)	C18—C19	1.494 (3)
C5—H5A	1.0065	C18—H18A	1.0133
C6—C7	1.494 (3)	C18—H18B	1.0133
C6—C8	1.513 (3)	C19—H19A	0.9857
C7—H7A	0.9614	C19—H19B	0.9857
C7—H7B	0.9614	C19—H19C	0.9857
			
C1—O1—C5	113.72 (15)	O4—C9—C10	108.9 (2)
C1—O2—C6	110.56 (16)	O5—C9—C10	108.8 (2)
C2—O3—C6	106.90 (15)	C11—C9—C10	111.7 (2)
C9—O4—C3	108.33 (17)	C9—C10—H10A	109.5
C9—O5—C4	110.03 (18)	C9—C10—H10B	109.5
C14—O6—C15	117.44 (19)	H10A—C10—H10B	109.5
C17—O8—C18	116.12 (19)	C9—C10—H10C	109.5
O1—C1—O2	110.33 (18)	H10A—C10—H10C	109.5
O1—C1—C2	115.16 (18)	H10B—C10—H10C	109.5
O2—C1—C2	103.64 (15)	C9—C11—H11A	109.5
O1—C1—H1A	109.2	C9—C11—H11B	109.5
O2—C1—H1A	109.2	H11A—C11—H11B	109.5
C2—C1—H1A	109.2	C9—C11—H11C	109.5
O3—C2—C3	108.38 (19)	H11A—C11—H11C	109.5
O3—C2—C1	104.60 (18)	H11B—C11—H11C	109.5
C3—C2—C1	114.14 (18)	C5—C12—C13	112.06 (17)
O3—C2—H2A	109.8	C5—C12—H12A	109.2
C3—C2—H2A	109.8	C13—C12—H12A	109.2
C1—C2—H2A	109.8	C5—C12—H12B	109.2
O4—C3—C2	107.09 (19)	C13—C12—H12B	109.2
O4—C3—C4	104.26 (18)	H12A—C12—H12B	107.9
C2—C3—C4	114.1 (2)	C14—C13—C17	108.06 (19)
O4—C3—H3A	110.4	C14—C13—C12	112.71 (17)
C2—C3—H3A	110.4	C17—C13—C12	112.42 (17)
C4—C3—H3A	110.4	C14—C13—H13A	107.8
O5—C4—C5	109.92 (18)	C17—C13—H13A	107.8
O5—C4—C3	104.44 (18)	C12—C13—H13A	107.8
C5—C4—C3	113.11 (19)	O7—C14—O6	124.5 (2)
O5—C4—H4A	109.7	O7—C14—C13	126.2 (2)
C5—C4—H4A	109.7	O6—C14—C13	109.2 (2)
C3—C4—H4A	109.7	O6—C15—C16	107.3 (2)
O1—C5—C4	109.26 (18)	O6—C15—H15A	110.2
O1—C5—C12	107.79 (17)	C16—C15—H15A	110.2
C4—C5—C12	113.28 (17)	O6—C15—H15B	110.2
O1—C5—H5A	108.8	C16—C15—H15B	110.2
C4—C5—H5A	108.8	H15A—C15—H15B	108.5
C12—C5—H5A	108.8	C15—C16—H16A	109.5
O3—C6—O2	105.14 (15)	C15—C16—H16B	109.5
O3—C6—C7	109.3 (2)	H16A—C16—H16B	109.5
O2—C6—C7	109.6 (2)	C15—C16—H16C	109.5
O3—C6—C8	111.2 (2)	H16A—C16—H16C	109.5
O2—C6—C8	109.00 (19)	H16B—C16—H16C	109.5
C7—C6—C8	112.36 (18)	O9—C17—O8	123.5 (2)
C6—C7—H7A	109.5	O9—C17—C13	124.7 (2)
C6—C7—H7B	109.5	O8—C17—C13	111.8 (2)
H7A—C7—H7B	109.5	O8—C18—C19	108.2 (2)
C6—C7—H7C	109.5	O8—C18—H18A	110.1
H7A—C7—H7C	109.5	C19—C18—H18A	110.1
H7B—C7—H7C	109.5	O8—C18—H18B	110.1
C6—C8—H8A	109.5	C19—C18—H18B	110.1
C6—C8—H8B	109.5	H18A—C18—H18B	108.4
H8A—C8—H8B	109.5	C18—C19—H19A	109.5
C6—C8—H8C	109.5	C18—C19—H19B	109.5
H8A—C8—H8C	109.5	H19A—C19—H19B	109.5
H8B—C8—H8C	109.5	C18—C19—H19C	109.5
O4—C9—O5	106.31 (17)	H19A—C19—H19C	109.5
O4—C9—C11	111.6 (2)	H19B—C19—H19C	109.5
O5—C9—C11	109.4 (2)		
			
C5—O1—C1—O2	78.7 (2)	C2—O3—C6—C7	−146.7 (2)
C5—O1—C1—C2	−38.2 (2)	C2—O3—C6—C8	88.7 (2)
C6—O2—C1—O1	−122.13 (19)	C1—O2—C6—O3	16.5 (3)
C6—O2—C1—C2	1.7 (3)	C1—O2—C6—C7	133.8 (2)
C6—O3—C2—C3	152.18 (19)	C1—O2—C6—C8	−102.8 (2)
C6—O3—C2—C1	30.0 (2)	C3—O4—C9—O5	−26.3 (2)
O1—C1—C2—O3	101.3 (2)	C3—O4—C9—C11	93.0 (3)
O2—C1—C2—O3	−19.2 (2)	C3—O4—C9—C10	−143.3 (2)
O1—C1—C2—C3	−16.9 (3)	C4—O5—C9—O4	17.1 (3)
O2—C1—C2—C3	−137.5 (2)	C4—O5—C9—C11	−103.5 (2)
C9—O4—C3—C2	145.74 (19)	C4—O5—C9—C10	134.2 (2)
C9—O4—C3—C4	24.4 (2)	O1—C5—C12—C13	51.9 (2)
O3—C2—C3—O4	169.21 (18)	C4—C5—C12—C13	172.91 (19)
C1—C2—C3—O4	−74.7 (3)	C5—C12—C13—C14	62.8 (2)
O3—C2—C3—C4	−76.0 (2)	C5—C12—C13—C17	−174.81 (18)
C1—C2—C3—C4	40.2 (3)	C15—O6—C14—O7	0.4 (4)
C9—O5—C4—C5	−123.7 (2)	C15—O6—C14—C13	−179.3 (2)
C9—O5—C4—C3	−2.1 (3)	C17—C13—C14—O7	−104.0 (3)
O4—C3—C4—O5	−13.5 (2)	C12—C13—C14—O7	20.9 (4)
C2—C3—C4—O5	−129.97 (19)	C17—C13—C14—O6	75.7 (3)
O4—C3—C4—C5	106.0 (2)	C12—C13—C14—O6	−159.4 (2)
C2—C3—C4—C5	−10.5 (3)	C14—O6—C15—C16	171.0 (2)
C1—O1—C5—C4	69.3 (2)	C18—O8—C17—O9	−0.5 (4)
C1—O1—C5—C12	−167.25 (15)	C18—O8—C17—C13	177.98 (19)
O5—C4—C5—O1	74.9 (2)	C14—C13—C17—O9	23.8 (3)
C3—C4—C5—O1	−41.4 (2)	C12—C13—C17—O9	−101.2 (3)
O5—C4—C5—C12	−45.3 (3)	C14—C13—C17—O8	−154.6 (2)
C3—C4—C5—C12	−161.60 (18)	C12—C13—C17—O8	80.4 (2)
C2—O3—C6—O2	−29.2 (3)	C17—O8—C18—C19	−175.6 (2)

### Hydrogen-bond geometry (Å, °)

**Table:**

*D*—H···*A*	*D*—H	H···*A*	*D*···*A*	*D*—H···*A*
C1—H1A···O9^i^	0.98	2.43	3.381 (3)	163
C5—H5A···O7	1.01	2.59	3.185 (3)	117
C8—H8B···O7^i^	1.03	2.56	3.577 (3)	169
C12—H12A···O5	1.02	2.42	2.811 (3)	102
C13—H13A···O1	0.96	2.43	2.814 (3)	103
C16—H16B···O1^ii^	0.99	2.51	3.422 (3)	153

Symmetry codes: (i) *x*−1, *y*, *z*; (ii) −*x*+1, *y*+1/2, −*z*+1/2.
